# A taxonomic revision of the genus *Sinotrisus* Yin & Li (Coleoptera, Staphylinidae, Pselaphinae)

**DOI:** 10.3897/zookeys.205.3362

**Published:** 2012-07-04

**Authors:** Zi-Wei Yin, Shûhei Nomura, Li-Zhen Li

**Affiliations:** 1 Department of Biology, College of Life and Environmental Sciences, Shanghai Normal University, 100 Guilin Road, Xuhui District, Shanghai 200234, P. R. China; 2 Department of Zoology, National Museum of Nature and Science, 3-23-1, Hyakunin-cho, Shinjuku-ku, Tokyo, 169-0073, Japan

**Keywords:** Coleoptera, Staphylinidae, Pselaphinae, *Sinotrisus*, China, Vietnam, revision

## Abstract

The genus *Sinotrisus* Yin & Li, comprising four species, is redefined and revised. Members of *Sinotrisus* are often found with ants of the subfamily Formicinae, or in humid forest habitats. The type speciesand three new species are (re-)described and illustrated: *Sinotrisus kishimotoi* Yin & Nomura, **sp. n.** (China: Sichuan), *Sinotrisus nomurai* Yin, Li & Zhao (type species) (China: Zhejing), *Sinotrisus sinensis* Yin & Nomura, **sp. n.** (China: Sichuan) and *Sinotrisus vietnamensis* Yin & Nomura, **sp. n.** (Vietnam: Lai Chau). A key is included as an aid to distinguishing these species.

## Introduction

Batrisitae (Newton & Thayer, 1995) comprises more than 220 genera distributed in all zoogeographical regions except for New Zealand, and about one-third of them are known from Asia (Newton and Chandler 1989 and subsequent papers). The recently established genus *Sinotrisus* Yin & Li currently contains a single species, *Sinotrisus nomurai* Yin, Li & Zhao, from East China (Yin et al. 2010), which is known from two males collected in a nest of the ant genus *Lasius*. A recent examination of the junior author’s pselaphine collection revealed three new *Sinotrisus* species from the Oriental region. The need of a generic redefinition of *Sinotrisus* arose immediately after the discovery of the new species. Thus, in this paper we redefine the genus, redescribe the type species, describe the new species and provide illustrations of major diagnostic features of all species. A key is provided to assist in the identification of these species.

The terminology used here is the same as that used by Chandler (2001) in his revision of the genera of Australian Pselaphinae, except we use ‘ventrite’ instead of ‘sternite’ when concerning the meso- and metathoracic structures.

A slash (/) is used to separate lines on the same label, and a double slash (//) is used to separate different labels on the same pin.

Measurements are in millimeters; the following acronyms are used in the text: AL–length of the abdomen along the midline; AW–maximum width of the abdomen; BL–length of the body (**=** HL+PL+EL+AL); EL–length of the elytra along the suture; EW–maximum width of the elytra; HL–length of the head from the anterior clypeal margin to the occipital constriction; HW–width of the head across eyes; PL–length of the pronotum along the midline; PW–maximum width of the pronotum.

Material treated in this study is housed in the following public institutions:

**SNUC** Insect Collection of Shanghai Normal University, Shanghai, P. R. China (Zi-Wei Yin);

**NSMT** National Museum of Nature and Science, Tokyo, Japan (Shûhei Nomura).

## Taxonomy

### Sinotrisus

Yin & Li, 2010

http://species-id.net/wiki/Sinotrisus

Sinotrisus Yin & Li, 2010: 249.

#### Type species.

*Sinotrisus nomurai* Yin, Li & Zhao, 2010 (by original monotypy).

#### Diagnosis.

Head trapezoidal; frontal rostrum low, antennal tubercles moderately raised. Pronotum with median and lateral longitudinal sulci; small antebasal spines present, lacking lateral spines; median longitudinal sulcus broadened posteriorly to form longitudinal impression, usually lacking median antebasal fovea in impression. Elytra with three basal fovea, discal striae shallow, extending to half elytral length. Tergite IV longest, with thick triangular ridge formed by inner and outer marginal carinae.

#### Redescription.

Length 3.0–3.3. Reddish brown. Head trapezoidal; with frontal rostrum low, antennal tubercles moderately prominent; with nude, deep vertexal foveae; occipital margins usually carinate; postocular margins narrowing toward head base; with eleven antennomeres, clubs weakly to distinctly indicated by apical three antennomeres, ocular-mandibular carinae present; eyes roundly prominent; maxillary palpomeres III triangular, IV narrowed to base in basal half; gular carina present; foveae close in large pit.

Pronotum with distinct lateral longitudinal sulci, median longitudinal sulcus ending posteriorly as broader longitudinal antebasal impression, then followed by short median carina; lateral antebasal foveae distinct; antebasal spines minute or absent, small spines variably present along discal ridges; lateral margins lacking spines; with both inner and outer pair of basolateral foveae present; paranotal carinae at least extending anteriorly to half prosternal length; lateral procoxal foveae present.

Each elytron with three distinct basal foveae, shallow discal stria extending to half elytral length; with complete sutural and marginal striae. Thorax with lateral mesoventral foveae forked, median mesoventral foveae with openings touching, into shared transverse cavity; with large mesocoxal foveae; lateral metaventral foveae present; metaventrite with narrow posteromedian notch. Legs with second and third tarsomeres subsequent in length.

Tergite IV longer than subsequent one, with inner marginal carinae extending entire tergal length, together with outer marginal carinae forming thick triangular ridge; mediobasal sulcus deep between mediobasal foveae, sulcus bracketed by short, tuberculate discal carinae; lateral foveae at mesal and lateral margins of short, deep basolateral sulci; tergite V with thin marginal carinae, punctiform mediobasal and basolateral foveae present; VI with marginal carinae indistinct, mediobasal and inner pair of basolateral foveae as shallow trace; VII with one pair of basolateral foveae and minute lateral tubercles. Sternite IV about twice length of V at midline, with large mediobasal and two pairs of small basolateral foveae; sternites V–VII each with one pair of basolateral foveae. Foveae of abdominal segments V–VII often overlapped by previous segment.

Males with vertex, apices of mesotibiae and metatrochanters modified. Aedeagus with basal bulb greatly constricted basally; paramere fused to median lobe to form ventral lobe; articulated dorsal lobe offset to right side.

#### Comparative notes.

The genus ismorphologically similar to *Batrisodes* Reitter of the *Batrisus* genus-group, but does not fit any subgeneric concept *sensu* Park (1951). *Sinotrisus* is here placed as a member of *Tribasodes* group by the males having protuberant metatrochanters and the aedeagus with an articulated dorsal lobe (genus-groups *sensu* Nomura and Idris 2003). The large genus *Batrisodes* holds many Asian species described by Raffray (1894) and Jeannel (1958), but at least some of these need to be re-examined and likely will be moved to other genera of the *Tribasodes* group (Nomura and Idris 2003; Nomura 2007). *Sinotrisus* shares with *Intestinarius* Kurbatov, *Dendrolasiophilus* Nomura and *Majappia* Nomura of the *Tribasodes* group the lack of the pronotal lateral spines. *Intestinarius* was included in the genus *Batrisodes*, but was later treated as a separate genus (Kurbatov 2007). Members of this genus have the head bearing three longitudinal sulci and the pronotum bearing five similar sulci, and have the aedeagus with numerous hairs at the apex of the ventral lobe. *Dendrolasiophilus* and *Majappia* seem to form a smaller group by the derived loss of characters, specifically the absence of sulci on the pronotum and the frequent loss of basal elytral foveae. *Dendrolasiophilus* has one basal elytral fovea, and lacks elytral discal striae; *Majappia* has the vertexal foveae connected by a transverse sulcus, and completely lacks basal foveae on the elytra. *Sinotrisus* also shares with *Hingstoniella* the constriction of the basal portion of the aedeagus and the similar placement of the male sexual features, but the broadly triangular pronotum lacking antebasal tubercles and foveae, the presence of a large basal elytral fovea, and the lack of carinae on the margins of tergites V–VI in *Hingstoniella* readily separate it from *Sinotrisus*.

### Sinotrisus kishimotoi

Yin & Nomura sp. n.

urn:lsid:zoobank.org:act:7432E342-C194-4D16-9139-DD82481DD409

http://species-id.net/wiki/Sinotrisus_kishimotoi

Figs 1 –2

#### Type material.

Holotype, male, labeled ‘Huangmaogeng Yakou / (2,710 m). Tianxi Xiang / Meigu Xian // [Sichuan, China] / [same locality data in Chinese] / 5.x.1997, T. kishimoto // HOLOTYPE [red] / *Sinotrisus kishimotoi* sp. n. / Yin and Nomura / det. 2012, NSMT’. Paratypes, 3 females, same label data as holotype, all bearing the following label: ‘PARATYPE [yellow] / *Sinotrisus kishimotoi* sp. n. / Yin and Nomura / det. 2012, NSMT’.

#### Diagnosis.

Vertex strongly modified in male. Antennomeres VII slightly elongate. Pronotum with minute spines along discal ridges; basolateral foveae small. Mesotibiae with apical spur longer than first tarsomeres.

#### Description.

**Figure 1.**
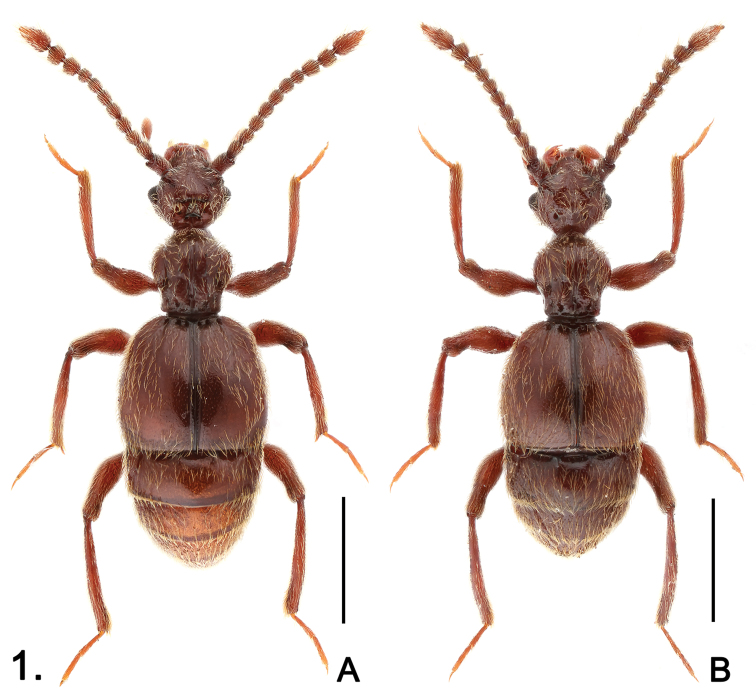
Habitus of *Sinotrisus kishimotoi* **A** male **B** female. Scales (mm): 1.0.

Male (Fig. 1A). Length 3.07. Head (Fig. 2A) slightly wider than long, HL 0.58, HW 0.65; vertex with deep cavity surrounding median triangular rostrum, anterior margin of cavity and posterior margin of rostrum edged by thickened ridge, cavity with tufts of setae at anterolateral margins, surface of rostrum densely setose, rostrum followed by short median carina; each eye composed of about 60 facets; lacking obvious occipital carinae; postocular margins evenly narrowed toward head base; antennomeres IV (Fig. 2B) subequal in length to VI, IX–XI enlarged to form distinct club. Pronotum about as long as wide, PL 0.64, PW 0.63; small median antebasal fovea in fusiform mediobasal impression; paranotal carinae extending half length of prosternum. Elytra slightly wider than long, EL 1.01, EW 1.16; slightly angulate at humeri. Mesotrochanters (Fig. 2C) with short, thick ventral spine; mesotibiae with apical spur (Fig. 2D) longer than first tarsomeres; metatrochanters (Fig. 2E) with broad, blunt elongate protuberance at ventral side. Abdomen wider than long, AL 0.84, AW 1.03; sternites IV with small triangular mediobasal ridge, V–VII with such ridge successively smaller. Aedeagus (Figs 2F–I) well-sclerotized, length 0.38.

Female (Fig. 1B). Similar to male in general, vertex and legs not modified. BL 3.05–3.12, HL 0.59–0.60, HW 0.67–0.68, PL 0.66–0.67, PW 0.64–0.66, EL 0.97–0.99, EW 1.16–1.18, AL 0.83–0.86, AW 1.07–1.10. Each eye composed of about 55 facets. Genital complex well-sclerotized (Figs 2J–K).

**Figure:**
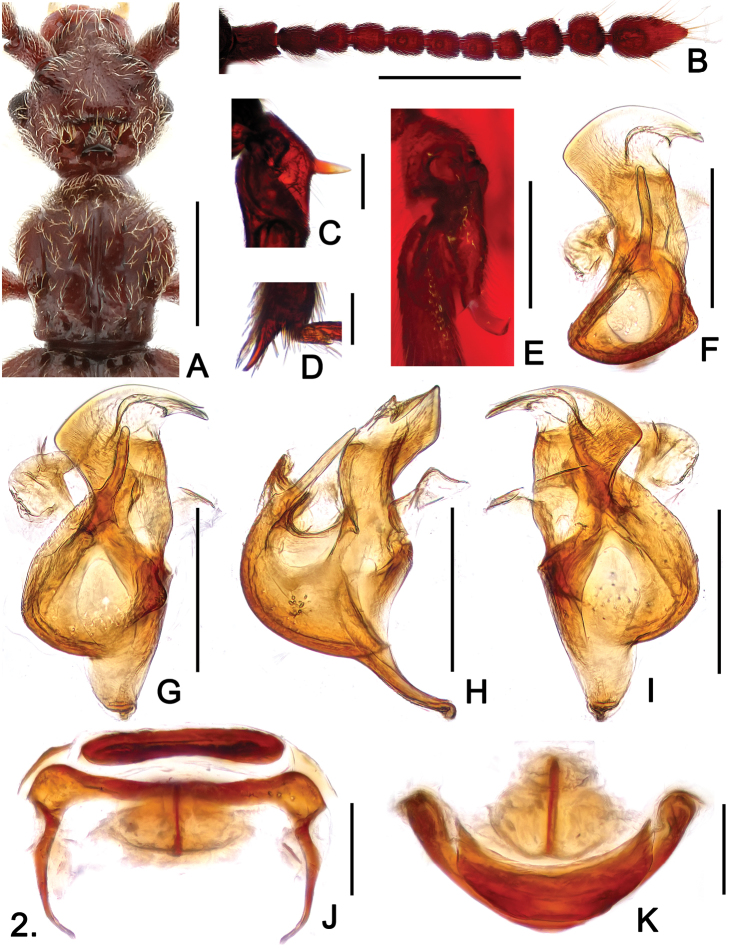
**Figure 2.** *Sinotrisus kishimotoi* **A** head and pronotum **B** antenna. **C** mesotrochanter **D** apex of mesotibia **E** metatrochanter **F** aedeagus, in top view **G** same, in dorsal view **H** same, in lateral view. **I** same, in ventral view **J** female genital complex, in dorsal view **K** same, in top view. Scales (mm): **A**, **B** = 0.5; **C**, **D**, **E**, **J**, **K** = 0.1; **F**, **G**, **H**, **I** = 0.2.

#### Distribution.

SouthwestChina: Sichuan.

#### Host ant.

*Formica* sp.

#### Etymology.

Named after the collector of the holotype, T. kishimoto.

### Sinotrisus nomurai

Yin, Li & Zhao

http://species-id.net/wiki/Sinotrisus_nomurai

Fig. 3

Sinotrisus nomurai Yin, Li & Zhao, 2010: 251.

#### Type material examined.

Holotype, male, labeled ‘China: Zhejiang Prov. / W. Tianmushan Mt. / firebreak / 01.v.2009, 1,400 m / Xiao-Bing SONG leg. // HOLOTYPE [red] / *Sinotrisus nomurai* sp. n. / Yin and Li / det. 2010, SNUC’. Paratype, male, same label data as holotype, with the following label: ‘PARATYPE [yellow] / *Sinotrisus nomurai* sp. n. / Yin and Li / det. 2012, SNUC’.

#### Diagnosis.

Vertex modified in the male. Antennomeres VII transverse. Pronotum lacking spines along discal ridges; basolateral foveae broad. Mesotibiae with one tiny and one long apical spur.

#### Redescription.

**Figure 3.**
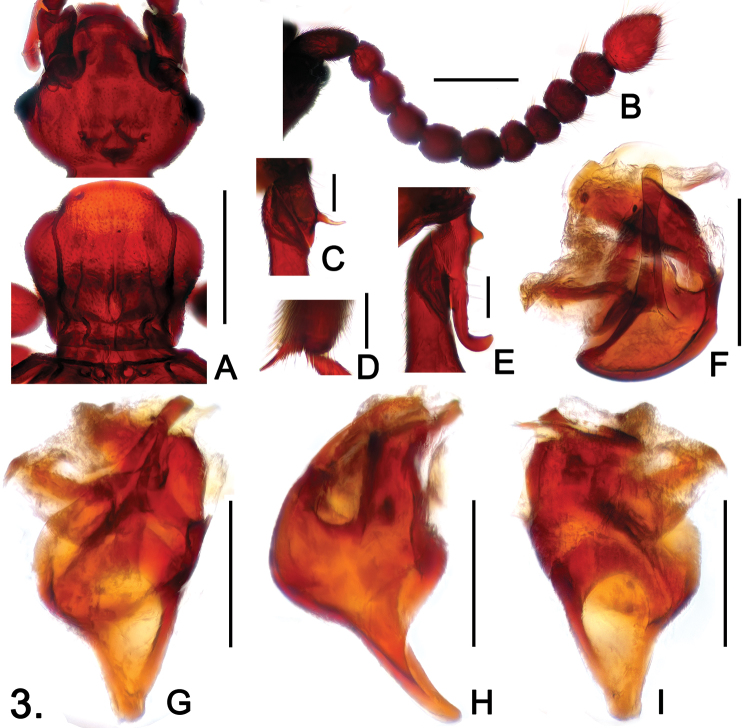
*Sinotrisus nomurai* **A** Head and pronotum **B** antenna **C** mesotrochanter **D** apex of mesotibia **E** metatrochanter **F** aedeagus, in top view **G** same, in dorsal view **H** same, in lateral view **I** same, in ventral view. Scales (mm): **A** = 0.5; **B** = 0.3; **C**, **D**, **E** = 0.1; **F**, **G**, **H**, **I** = 0.2.

Male. Length 3.30. Head (Fig. 3A) wider than long, HL 0.57, HW 0.71; surface convex anterior and posterior to vertexal foveae, forming median angulate projection; with distinct occipital carinae; postocular margins evenly narrowed toward head base; antennomeres IV (Fig. 3B) smaller than VI, IX–X transverse, XI nearly oval, clubs indistinct. Pronotum about as long as wide, PL 0.66, PW 0.68; mediobasal impression longitudinally oval, lacking median antebasal fovea; paranotal carinae extending through length of prosternum. Elytra slightly wider than long, EL 1.06, EW 1.18; angulate at humeri. Mesotrochanters (Fig. 3C) with long ventral spine slightly curved toward base in apical half; mesotibiae with apical spur (Fig. 3D) longer than first tarsomeres; metatrochanters (Fig. 3E) with blunt triangular tubercle and large elongate protuberance at ventral side. Abdomen slightly wider than long, AL 0.96, AW 1.08; tergites and sternites V–VII with basolateral ridges successively shorter and thinner. Aedeagus (Figs 3F–I) well-sclerotized, length 0.42.

Female. Unknown.

#### Distribution.

East China: Zhejiang.

#### Host ant.

*Lasius* sp.

### Sinotrisus sinensis

Yin & Nomura sp. n.

urn:lsid:zoobank.org:act:5536F78C-5ECA-4399-AFA8-FF78B62E1E59

http://species-id.net/wiki/Sinotrisus_sinensis

Figs 4A , 5

#### Type material.

Holotype, male, labeled ‘Majiagou (3,140 m) / Kangding Xian / [Sichuan, China] / [same locality data in Chinese] / 9.ix.1998 / Toshio Kishimoto leg. // HOLOTYPE [red] / *Sinotrisus sinensis* sp. n. / Yin and Nomura / det. 2012, NSMT’.

#### Diagnosis.

Vertex modified in male. Antennomeres VII elongate. Pronotum with indistinct spines along discal ridges; basolateral foveae punctiform. Mesotibiae with one tiny and another larger apical spur, larger spur shorter than first tarsomeres.

#### Description.

**Figure 4.**
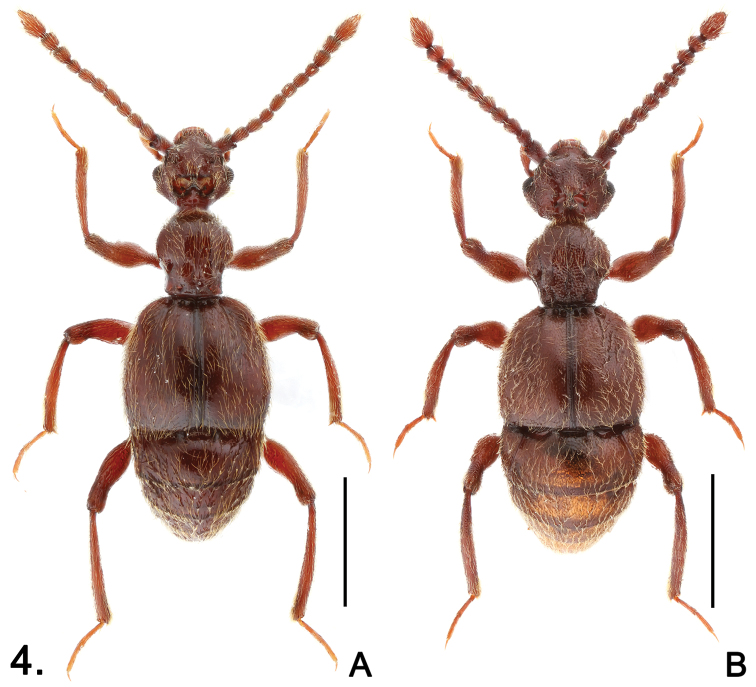
Habitus of *Sinotrisus*. **A** *Sinotrisus sinensis* **B** *Sinotrisus vietnamensis*. Scales (mm): 1.0

**Figure 5.**
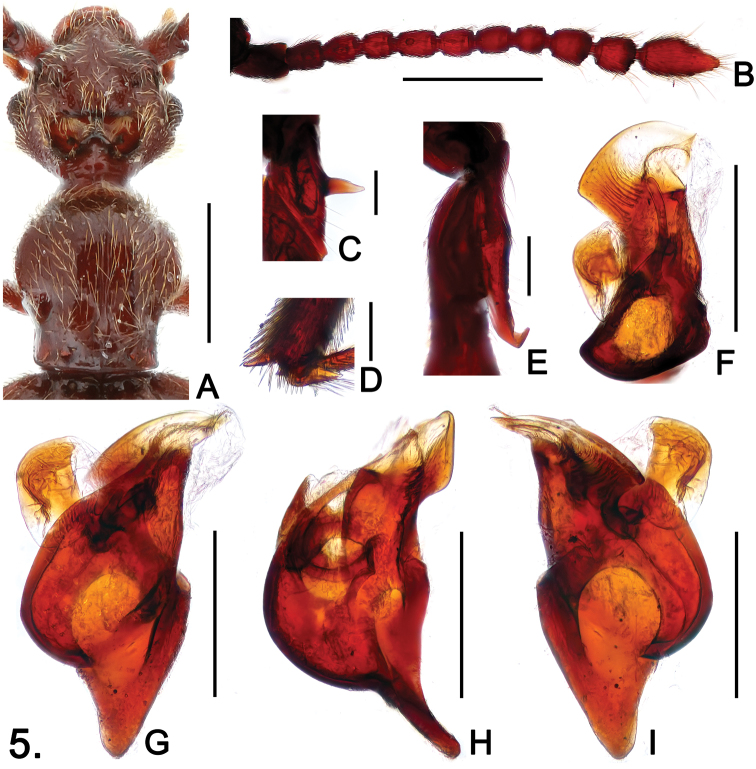
*Sinotrisus sinensis* **A** head and pronotum **B** antenna **C** mesotrochanter **D** apex of mesotibia **E** metatrochanter **F** aedeagus, in top view **G** same, in dorsal view **H** same, in lateral view **I** same, in ventral view. Scales (mm): **A**, **B** = 0.5; **C**, **D**, **E** = 0.1; **F**, **G**, **H**, **I** = 0.2.

Male (Fig. 4A). Length 3.20. Head (Fig. 5A) wider than long, HL 0.61, HW 0.66; vertex with foveae in broad median ‘∞’-shaped cavity, cavity densely setose at anterior margin; lacking occipital carinae; postocular margins parallel for short distance beneath eyes, then evenly narrowed toward head base; antennomeres IV (Fig. 5B) slightly shorter than VI, clubs formed by apical three antennomeres, IX–X nearly quadrate, XI elongate, narrowed toward apex in apical half. Pronotum slightly longer than wide, PL 0.65, PW 0.62; median longitudinal sulcus slightly widened to form oval antebasal impression, lacking median antebasal fovea; paranotal carinae extending through length of prosternum. Elytra slightly wider than long, EL 1.02, EW 1.17; slightly angulate at humeri. Mesotrochanters (Fig. 5C) with thick, short ventral spine; mesotibiae with apical spur (Fig. 5D) shorter than first tarsomeres; metatrochanters (Fig. 5E) with large elongate ventral projection. Abdomen slightly wider than long, AL 0.92, AW 1.05; sternites IV–VI with triangular mediobasal and basolateral ridges successively shorter and thinner. Aedeagus (Figs 5F–I) well-sclerotized, length 0.41.

Female. Unknown.

#### Distribution.

Southwest China: Sichuan.

#### Host ant.

*Formica* sp.

#### Etymology.

Named after the country where the type locality lies.

### Sinotrisus vietnamensis

Yin & Nomura sp. n.

urn:lsid:zoobank.org:act:0E6979F9-99FC-408B-BBF3-038C82E92618

http://species-id.net/wiki/Sinotrisus_vietnamensis

Figs 4B , 6

#### Type material.

Holotype, male, labeled ‘Mt. Phang Si Pang (moss: / 2,000 m). Lai Chau Prov. / [N. VIETNAM] / 17.v.2003, S. Nomura leg. // HOLOTYPE [red] / *Sinotrisus vietnamensis* sp. n. / Yin and Nomura / det. 2012, NSMT’.

#### Diagnosis.

Vertex modified in male. Antennomeres VII greatly transverse. Pronotum lacking spines along discal ridges; basolateral foveae small. Mesotibiae with apical spur about as long as first tarsomeres.

#### Description.

**Figure 6.**
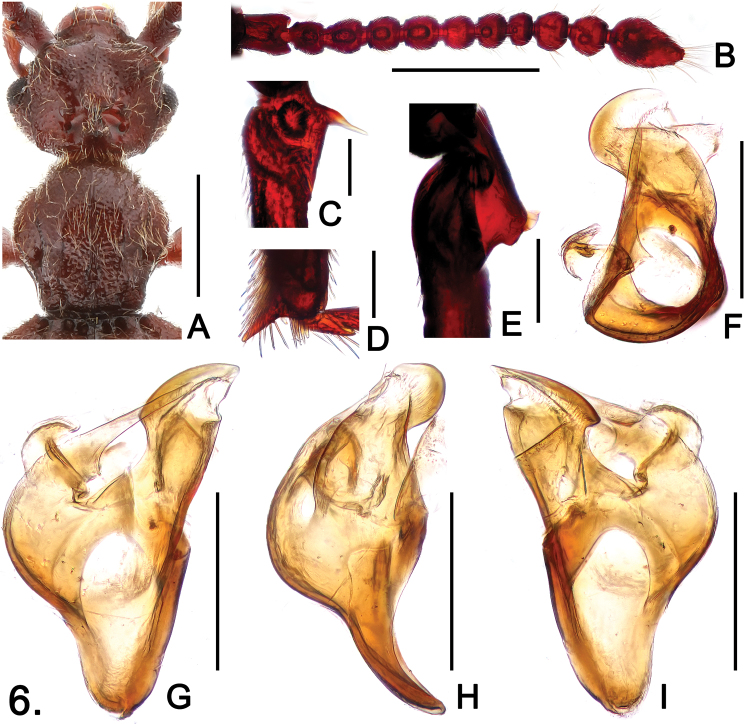
*Sinotrisus vietnamensis* **A** head and pronotum **B** antenna **C** mesotrochanter **D** apex of mesotibia **E** metatrochanter **F** aedeagus, in top view **G** same, in dorsal view **H** same, in lateral view **I** same, in ventral view. Scales (mm): **A**, **B** = 0.5; **C**, **D**, **E** = 0.1; **F**, **G**, **H**, **I** = 0.2.

Male (Fig. 4B). Length 3.00. Head (Fig. 6A) distinctly wider than long, HL 0.57, HW 0.71; frons convex medially, extending posteriorly to anterior margin of vertexal cavity; shallow transverse cavity with large foveae, area posterior to cavity strongly raised medially to form rostrum, densely covered with setae; with distinct occipital carinae; postocular margins evenly narrowed toward head base; antennomeres IV (Fig. 6B) about same length as VI, clubs formed by apical three antennomeres, IX strongly transverse, X slightly longer than IX, XI nearly oval, narrowed toward apex in apical half. Pronotum about as long as wide, PL 0.63, PW 0.65; median longitudinal sulcus slightly widened posteriorly to form shallow oval antebasal impression, lacking median antebasal fovea; paranotal carinae extending through length of prosternum. Elytra slightly wider than long, EL 0.92, EW 1.13; barely angulate at humeri. Mesotrochanters (Fig. 6C) with long basoventral spine; mesotibiae with apical spur (Fig. 5D) about same length of first tarsomeres; metatrochanters (Fig. 6E) broadly expanded ventrally, with small blunt tubercle at ventral margin. Abdomen slightly wider than long, AL 0.88, AW 1.10; segments lacking basal ridges. Aedeagus (Figs 6F–I) well-sclerotized, length 0.39.

Female. Unknown.

#### Distribution.

Vietnam: Lai Chau.

#### Habitat.

The individual was collected from a pile of moist moss.

#### Etymology.

Named after the country where the type locality lies.

### Key to males

**Table:** 

1	Antennomeres VII strongly transverse (Figs 3B, 6B)	2
–	Antennomeres VII slightly to moderately elongate (Figs 2B, 5B)	3
2	Antennomeres VI slightly larger than V (Fig. 3B); metatrochanters with small triangular spine and large elongate protuberant at ventral margin (Fig. 3E); ventral spine of mesotrochanters curved basally in apical half (Fig. 3C). (China: Zhejiang)	*Sinotrisus nomurai* Yin, Li & Zhao
–	Antennomeres VI slightly smaller than V (Fig. 6B); metatrochanters broadly expended ventrally, with small, blunt ventral tubercle (Fig. 6E); ventral spine of mesotrochanters not curved in apical half (Fig. 6C). (Vietnam: Lai Chau)	*Sinotrisus vietnamensis* sp. n.
3	Postocular margins parallel for a short distance beneath eyes, then straightly narrowed toward head base (Fig. 5A); pronotum lacking spines along discal carinae (Fig. 5A); mesotibiae with apical spur shorter than first tarsomere (Fig. 5D). (China: Sichuan)	*Sinotrisus sinensis* sp. n.
–	Postocular margins evenly narrowed toward head base (Fig. 2A); pronotum with two pairs of minute spines along discal carinae (Fig. 2A); mesotibiae with apical spur longer than first tarsomere (Fig. 2D). (China: Sichuan)	*Sinotrisus kishimotoi* sp. n.

## Supplementary Material

XML Treatment for Sinotrisus

XML Treatment for Sinotrisus kishimotoi

XML Treatment for Sinotrisus nomurai

XML Treatment for Sinotrisus sinensis

XML Treatment for Sinotrisus vietnamensis
